# Ephrin-B1 regulates cell surface residency of heparan sulfate proteoglycans (HSPGs) and complexes with the HSPG CD44V3–10 and fibroblast growth factor receptors

**DOI:** 10.1093/glycob/cwaf020

**Published:** 2025-04-28

**Authors:** Kristian Prydz, Roger Simm, Erna Davydova, Hans-Christian Aasheim

**Affiliations:** Section for Physiology and Cell Biology, Department of Biosciences, University of Oslo, PO Box 1066 Blindern, 0316 Oslo, Norway; Section for Genetics and Evolutionary Biology, Department of Biosciences, University of Oslo, PO Box 1066 Blindern, 0316 Oslo, Norway; Section for Physiology and Cell Biology, Department of Biosciences, University of Oslo, PO Box 1066 Blindern, 0316 Oslo, Norway; School of Health Sciences, University College Kristiania, 0153 Oslo, Norway

**Keywords:** CD44, Eph receptors, Ephrin-B1, FGF receptors, heparan sulfate proteoglycans

## Abstract

The ephrin family of membrane proteins mediate intracellular signalling as ligands of transmembrane Eph tyrosine kinase receptors during cell–cell interactions. Ephrin/Eph signalling regulates processes like cell migration and angiogenesis and is of particular importance during embryonic development. Ephrins-A3 and -B3 can also bind to cell surface-associated and soluble heparan sulfate proteoglycans (HSPGs) that also play important roles during early development. Here we show that ephrins-B1, -B2, and -B3 all can bind in *cis* to cell surface HSPGs, while only ephrin-B1 interacts with cell surface HSPGs in a way that retards HSPG endocytosis. Expressing ephrin-B1 in HEK293T cells, using polyethyleneimine (PEI) as transfection agent, increased cell surface levels of HSPGs which were detected by an anti-heparan sulfate (HS) antibody or by ephrin-B3-Fc binding. Ephrin-B1 in the plasma membrane seemed to retard PEI-induced HSPG internalisation and degradation. Binding of HSPGs by ephrin-B1 was observed for the human, mouse, xenopus, and zebrafish homologs, and did not require the cytoplasmic tail of ephrin-B1 that contains tyrosines shown to be involved in intracellular signalling. Furthermore, ephrin-B1 could bind the HSPG variant of CD44 (CD44V3–10), a complex that could further associate with fibroblast growth factor receptors (1 and 4) after co-expression with one of these receptors. In summary, our data indicate that ephrin-B1 can regulate cellular HSPG turnover and is able to form complexes of potential biological importance with CD44V3–10 and fibroblast growth factor receptors.

## Introduction

The ephrin family of cell surface glycoproteins comprises eight members separated into two sub-families; ephrin-As (A1-A5) that are linked to the plasma membrane via a glycosylphosphatidylinositol (GPI) anchor and ephrin-Bs (B1-B3) that are transmembrane proteins. Generally, ephrins bind to corresponding Eph transmembrane receptor tyrosine kinases (EphAs and EphBs) on adjacent cells to induce intracellular signalling (Kullander and Klein 2002; Kania and Klein 2016). The intracellular domain of the ephrin-B proteins is highly conserved and contains a PDZ binding motif (Lin et al. 1999) and tyrosines that become phosphorylated upon Eph receptor interaction to engage in bidirectional intracellular signalling (Wilkinson 2014). Eph receptors, ephrins, and their involvement in cell–cell interactions have been observed in early metazoans—even in sponges, but reverse signalling via transmembrane ephrins seems to be a later evolutionary event (Schwieterman et al. 2016; Arcas et al. 2019).

In mammals, ephrin-B1 is widely expressed and plays important roles during development (Cayuso et al. 2016; Yoon et al. 2021) and in a variety of tissues in adults. Mutations in the gene encoding ephrin-B1 give rise to Craniofrontonasal syndrome (Twigg et al. 2004; Toral-Lopez et al. 2016), and knock-out of ephrin-B1 in mice results in skeletal defects (Nguyen et al. 2016), suggesting an important role in skeletal development (Compagni et al. 2003). Ephrin-B1 expressed in astrocytes controls synapse formation in the hippocampus (Koeppen et al. 2018) and is together with the other ephrin-Bs generally required for synapse function (Henderson and Dalva 2018) and maintenance and migration of interneurons (Talebian et al. 2017). Ephrin-B1 is also a specific marker of mature germinal center-resident B-cells, mediating their retention within germinal centers (Laidlaw et al. 2017) and at the same time preventing B-cell binding to T-cells by ephrin-B1-EphB6 mediated repulsion (Lu et al. 2017). Eph/ephrin based repulsion mechanisms are also important for neurite outgrowth and axon guidance (Kullander and Klein 2002; Sullivan and Bashaw 2023). Exosomes released from different cell types, including neuronal cells, contain ephrins and Eph receptors, and exosomes harbouring EphB2 bind to and are internalised by ephrin-B1 positive cells and may in this way induce signalling and growth cone collapse (Gong et al. 2016). Cell surface Eph/ephrins can be removed from the plasma membrane by traditional endocytic mechanisms, but in addition by trans-endocytosis (Marston et al. 2003; Pitulescu and Adams 2010), also called trogocytosis (Gong et al. 2019), or by proteolytic shedding to the extracellular environment (Tomita et al. 2006; Pitulescu and Adams 2010). Shed ephrin molecules as such do not seem to participate in signalling processes, since these are reported to require receptor multimers in the plasma membrane (Schaupp et al. 2014). Higher order clusters of ephrins and Eph receptors engaged in signalling may form via homooligomerization or by formation of heterooligomers (Himanen et al. 2010; Janes et al. 2011; Schaupp et al. 2014), where the actual receptor(s) involved determine the oligomerization tendency (Seiderake et al. 2013). Receptor dimers interacting with their ephrin ligands are more likely to be removed from the cell surface by shedding or internalisation, which again can lead to collapse of cell protrusions, while higher-order clusters engage more consistently in signalling domains (Pitulescu and Adams 2010; Janes et al. 2011; Seiderake et al. 2013; Schaupp et al. 2014). Collapse and repulsion mechanisms can be influenced by other molecules in the plasma membrane. Activation of fibroblast growth factor receptor 1 (FGFR1) in cells expressing EphB2 inhibits cellular segregation, repulsion, and collapse induced by ephrin-B1 phosphorylation (Lee et al. 2009) and stimulation of EphB2 (Poliakov et al. 2004). During wound healing, an early event is the increased expression of ephrin-B1 and the induced expression of the EphB2 receptor in basal keratinocytes. This leads to downregulation of adherens and tight junction proteins in epidermal cells near the wound, allowing these cells to migrate (Nunan et al. 2015).

Overexpression of ephrins and Eph receptors has been observed in a wide variety of tumour types (Surawska et al. 2004; Pasquale 2008; Liu et al. 2022). Dysregulation of ephrin-B1 expression is reported in several cancers. Ephrin-B1 is expressed in a subset of human osteosarcoma tumours, and clinical data indicate this expression to correlate with a more aggressive malignancy (Varelias et al. 2002). Ephrin-B1 is also upregulated in ovarian carcinomas (Castellvi et al. 2006) and in glioblastomas (Nakada et al. 2010). Ephrin-B1 is differentially expressed in different subgroups of medulloblastoma, where its overexpression decreases adhesion and promotes proliferation (McKinney et al. 2015).

Although ephrin-B members can bind Eph receptors at the surface of opposing cells, they may also associate with other proteins in *cis*—in the same cell membrane, and with a variety of cytoplasmic proteins (Brückner et al. 1999; Lin et al. 1999; Bong et al. 2004; Bong et al. 2007; Yoon et al. 2021; Cecchini and Cornelison 2022). A study in *Xenopus* showed that activated FGFR could associate with ephrin-B1 (Chong et al. 2000). FGFRs have been shown to induce phosphorylation of the cytoplasmic domain of ephrin-B1, which prevents interaction with Dishevelled (Lee et al. 2009). Another study showed that claudins, major constituents of tight junctions, associate laterally with ephrin-B1 molecules in common cell surface domains (Tanaka et al. 2005). Both ephrin-B1 and ephrin-B2 have been shown to associate with the interleukin-7 receptor α, retarding internalization of the receptor from the cell surface (Luo et al. 2011). The secreted glycoprotein Reelin, that is involved in guidance of neurons, has also been shown to be associated with ephrin-B family members (Sentürk et al. 2011). A recent study, using a combination of proximity labeling and single molecule binding assays to discover E-cadherin interacting proteins, showed that E-cadherin and ephrin-B1 can interact in in vitro assays (Shafraz et al. 2020). Ephrin-B2 and ephrin-B3 are also entry receptors for Nipah and Hendra viruses (Negrete et al. 2006; Bossart et al. 2008; Xu et al. 2012). Binding of these viruses at the cell surface has been shown to involve HS and this binding can be blocked by heparin (Mathieu et al. 2015). Ephrin-B1 and ephrin-B2, but not ephrin-B3, were recently shown to bind Cedar virus, a related nonpathogenic henipavirus (Pryce et al. 2019).

Proteoglycans (PGs) are protein cores that enter the secretory pathway in the endoplasmic reticulum and acquire glycosaminoglycan (GAG) chains when passing through the Golgi apparatus (Prydz and Dalen 2000; Prydz 2015). Arriving at the plasma membrane, PGs are either secreted or remain cell associated. Secreted PGs are often associated with extracellular matrices, while cell attachment is via a transmembrane domain or a GPI anchor, typical for glypican family members. The GAG chains of PGs are highly negatively charged, due to glucuronic or iduronic acid sugars alternating in disaccharide units with GlcNAc (HS/heparin) or GalNAc (chondroitin sulfate/dermatan sulfate) in the polymerized GAG chains. In addition, the disaccharide units undergo sulfation in various positions and to a variable extent, contributing to the overall negative charge (Lindahl et al. 1998; Annaval et al. 2020). Cell surface PGs therefore attract protein domains, peptides and other types of molecules that are positively charged, and the sulfation pattern of the GAG chains may determine their binding specificity. Heparin is a highly sulfated variant of HS and can block most HS-ligand interactions. HSPGs are well-known co-receptors for growth factors like fibroblast growth factors (FGFs) and are required for proper signalling both in physiological and pathological processes (Hassan et al. 2021; Chen et al. 2023).

Polyethylenimine (PEI) is a much used transfection reagent (Huh et al. 2007). PEI has been shown to require HSPGs to mediate gene delivery (Payne et al. 2007), and certain HSPGs are rapidly endocytosed when PEI:DNA complexes are added to the cell medium (Paris et al. 2008; Mozley et al. 2014; Hess et al. 2017). PEI is also used to reveal the localization of HSPGs in microscopy studies (Sauren et al. 1994; De Andrea et al. 2011), thus binding of PEI to HSPGs is an established phenomenon as has also been shown for other small positively charged molecules (Belting et al. 1999). Certain ephrins and Eph receptors have been shown to interact with HSPGs (Irie et al. 2008; Holen et al. 2011; Prydz et al. 2018). In *C. elegans*, both the HSPGs syndecan-1 and the ephrin EFN-4 are required for neurite extension. The HS chains were involved, since knock-down of HS modifying enzymes inhibits ectopic neurite branching, but direct interaction between the ephrin and HS has not yet been shown (Schwieterman et al. 2016). Regarding the large number of potential interactions, a significant amount of work remains to clarify the mechanisms involved. Whether ephrins and/or Eph receptors regulate the function and turnover of HSPGs – and vice versa—has, however, not been addressed to any extent. We have previously shown that the extracellular part of ephrin-B3, lacking the transmembrane and cytoplasmic domains, binds to both extracellular and cell membrane-associated HSPGs, involving two positively charged residues, Arg178 and Lys179 (Holen et al. 2011; Prydz et al. 2018).

In this work we show that expression of ephrin-B1 in HEK293T cells increases the level of HSPGs in the cell membrane, most likely by preventing HSPG endocytosis induced by the transfection agent PEI, since we could not observe the same effect when transfection was performed with Lipofectamine. The interaction of ephrin-B1 with HSPGs seems to be in *cis* in the membrane, with ephrin-B1 and the HSPGs localized to the same membrane domains. When expressing the HSPG core protein variant of CD44 (CD44V3–10), this HSPG could be co-precipitated with ephrin-B1, to our knowledge the first report of an interaction between these two proteins. When co-expressing FGFRs 1 or 4 in addition to ephrin-B1 and CD44V3–10, all three proteins could be precipitated together. While ephrin-B1 has been shown to have the ability to interact with FGFRs (Chong et al. 2000; Moore et al. 2004; Poliakov et al. 2008; Lee et al. 2009), CD44 can interact with both FGF and the FGFRs (Nedvetzki et al. 2003; Zhen et al. 2018), but all three signaling proteins have not previously been shown to form a complex together.

## Results

### Expression of ephrin-B1 in HEK293T cells by PEI-mediated transfection increases cell surface HSPG levels

**Fig. 1:**
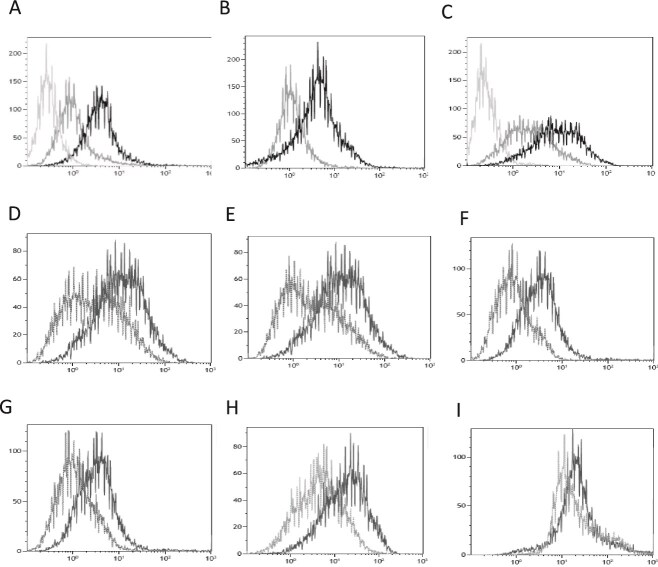
Flow cytometry analysis of cell surface HS in HEK293T cells transiently transfected using PEI (unless otherwise indicated) to express ephrins-B1, -B2, or -B3. A) Cells expressing ephrin-B1 (black) or mock transfected (grey) incubated with ephrin-B3-fc and PE-conjugated anti-human IgG (PE-IgG) 2nd layer. Light grey is 2nd layer control. B) Cells expressing ephrin-B1 incubated with heparin (grey) or not (black) followed by ephrin-B3-fc binding and PE-IgG. C) Cells expressing ephrin-B1 (black) or mock transfected (grey) incubated with anti-HS antibody followed by FITC-conjugated anti mouse IgM (FITC-IgM). Light grey 2nd layer control. D) Cells expressing ephrin-B1 (black) or ephrin-B2 (grey) incubated with anti-HS antibody and FITC-IgM. E) Cells expressing ephrin-B1 (black) or ephrin-B3 (grey) incubated with anti-HS antibody and FITC-IgM. F) Cells expressing ephrin-B1 (black) or ephrin-B2 (grey) incubated with ephrin-B3-fc and PE-IgG. G) Cells expressing ephrin-B1 (black) or ephrin-B3 (grey) incubated with ephrin-B3-fc and PE-IgG. H) Cells mock transfected using PEI (grey) or Lipofectamine (black) incubated with anti-HS antibody and FITC-IgM. I) Cells transfected using PEI (grey) or Lipofectamine (black) expressing ephrin-B1 incubated with EphB2-fc and PE-IgG.

We have previously shown (Holen et al. 2011; Prydz et al. 2018) that soluble ephrin-B3 (ephrin-B3-Fc) can bind to both secreted and cell-associated HSPGs. In the experiments reported here, we initially observed increased binding of soluble ephrin-B3-Fc fusion protein to HEK293T cells that had been transfected to express ephrin-B1 using the transfection agent PEI, when comparing to mock transfected cells (Fig. 1A). PEI and other transfection agents (Mislick and Baldeschwieler 1996) have previously been shown to bind to and induce endocytosis of HSPGs from the cell surface when PEI:DNA complexes are added to the cell culture medium (Payne et al. 2007; Paris et al. 2008; Mozley et al. 2014). This increased binding of ephrin-B3-Fc to HEK293T cells that expressed ephrin-B1 was blocked in the presence of heparin (Fig. 1B), indicating that the enhanced binding was HSPG-dependent. This was confirmed by investigating cell surface binding of the 10E4 antibody recognizing native HS chains (David et al. 1992). The flow cytometry diagram in Fig. 1C shows increased binding of the anti-HS antibody to PEI-transfected HEK293T cells expressing ephrin-B1. Binding experiments were also performed with HEK293T cells transfected to express ephrin-B2 or ephrin-B3 using the same PEI-based protocol, but neither the anti-HS antibody nor ephrin-B3-Fc bound more efficiently to HEK293T cells expressing ephrin-B2 or ephrin-B3 than to mock transfected cells (Fig. 1D-G). Similar results were obtained when HSPG was detected with either an anti-HS antibody (Fig. 1D and E) or ephrin-B3-Fc (Fig. 1F and G). PEI alone induced endocytosis of HSPGs, since PEI incubation of HEK293T cells lead to reduced anti-HS antibody binding when compared to cells incubated with the alternative transfection agent Lipofectamine (Fig. 1H). Lipofectamine incubation, or incubation with no agent gave the same result (data not shown). Similar expression levels of ephrin-B1 were observed when cells where transfected with ephrin-B1 cDNA using either PEI or Lipofectamine as shown by binding of EphB2-Fc, which binds ephrin-B1, but does not bind to HSPGs (Fig. 1I). The increase in cell surface HS binding was not a result of reduced HS shedding, since HS shedding was observed neither in untransfected cells nor in ephrin-B1 expressing cells (results not shown).

### Ephrin-B1 associates with sulfated macromolecules and is not a proteoglycan by itself

**Fig. 2:**
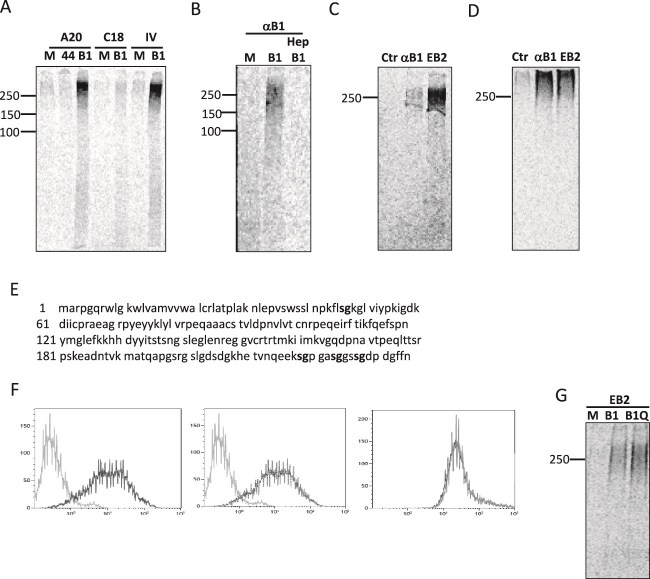
Co-precipitation of HSPGs and ephrin-B1. Ephrin-B1 is not an HSPG by itself. HEK293T cells PEI-transfected expressing CD44V3–10 (44), ephrin-B1 (B1) or mock (M), metabolically labelled with ^35^S-sulfate and lysed, incubated with anti-ephrin-B1 antibodies (A20, C18, or IV) and protein G beads before SDS-PAGE. (B) Mock (M) and ephrin-B1 (B1) expressing cells ^35^S-sulfate labelled and lysed. Lysates of cells expressing ephrin-B1 treated or not with Heparinase (hep) precipitated with anti-ephrin-B1 and protein G beads before SDS-PAGE. (C) Ephrin-B1-expressing cells ^35^S-sulfate labelled, lysed and precipitated with protein-G beads only (Ctr), anti-ephrin-B1 (α-B1), or EphB2-fc (EB2). (D) HT-29 cells ^35^S-sulfate labelled, lysed and precipitated with protein-G beads only (Ctr), anti-ephrin-B1 (α-B1), or EphB2-fc (EB2). (E) Primary sequence ephrin-B1 (extracellular part). Serine-glycines (SG) in bold. (F) Flow cytometry of HEK293T cells expressing ephrin-B1 (left panel) and ephrin-B1Q (middle panel) incubated with anti-HS and FITC-antibody 2nd layer (black). Grey: 2nd layer control. Right panel: Overlay of cells expressing ephrin-B1 (black) and ephrin-B1Q (grey) incubated with EphB2-fc and PE-antibody 2nd layer. (G) Cells mock transfected (M) or expressing ephrin-B1 (B1) or ephrin-B1Q (B1Q), ^35^S-sulfate labelled, washed, lysed, and precipitated with EphB2-fc bound to protein G beads.

The data presented in Fig. 1 indicated that expression of ephrin-B1 in HEK293T cells in the presence of the transfection agent PEI increased cell surface expression of HSPGs when comparing to mock transfected cells, either by affecting the synthesis or turnover of HSPGs, or alternatively because ephrin-B1 itself could be modified with HS chains in the Golgi apparatus underway to the cell surface. To distinguish these possibilities, HEK293T cells expressing ephrin-B1 were metabolically labelled with ^35^S-sulfate which is incorporated into HS GAG chains. Ephrin-B1 in lysates from these cells was immuno-isolated with different anti-ephrin-B1 antibodies. Precipitation with an anti-ephrin-B1 antibody (A20) from lysates of HEK293T cells expressing CD44V3–10 was used as negative control (Fig. 2A). To confirm that the metabolic label was associated with HS GAGs, lysates from ^35^S-sulfate-labelled cells were treated with heparitinase prior to precipitation with an anti-ephrin-B1 antibody. Heparitinase treatment removed the signal from the fraction of the precipitated ^35^S-sulfate-labelled macromolecules, demonstrating that the label was associated with HSPGs (Fig. 2B).

Next, we precipitated ^35^S-sulfate-labelled macromolecules from lysates of ephrin-B1 expressing HEK293T cells with the extracellular domain of the ephrin-B1 receptor EphB2-Fc. Also by this mode of precipitation HSPGs were isolated (Fig. 2C). Lastly, we performed both precipitation modes using either anti-ephrin-B1 or EphB2-Fc with lysates of ^35^S-sulfate-labelled HT29 cells, an epithelial cell line with endogenous expression of ephrin-B1 (Chiu et al. 2009). Both anti-ephrin-B1 and EphB2-Fc precipitated ^35^S-sulfated macromolecules from HT29 cell lysates (Fig. 2D).

HS GAG chains, chondroitin sulfate, dermatan sulfate and heparin GAGs, are linked to serines (S) followed by glycines (G) in SG sites in various PG protein cores. However, far from all potential SG modification sites in proteins passing through the secretory pathway in animal cells are utilized (Prydz and Dalen 2000; Prydz 2015). There are four SG motifs located in the extracellular part of the human ephrin-B1 protein sequence (Fig. 2E). The first SG site is located at amino acids 46–47, while the last three SGs are located among amino acids 218–228, near the trans-membrane domain of the protein. To investigate whether the serines in these SG sites are subject to HS modification and the reason for the increase in ^35^S-sulfate label in HEK293T cells expressing ephrin-B1, these serines HHwere altered to alanines by mutagenesis (ephrin-B1Q). HEK293T cells expressing ephrin-B1Q and wild type ephrin-B1 showed no difference in their capacity to bind the anti-HS antibody or EphB2-Fc by flow cytometry (Fig. 2F). The cells were also metabolically labelled with ^35^S-sulfate. EphB2-Fc precipitated sulfated macromolecules with similar efficiency from lysates of both cell lines (Fig. 2G). Thus, the metabolic label associated with precipitated ephrin-B1 is localized to HS-GAG chains attached to another protein core, and ephrin-B1 is not an HSPG by itself when expressed in HEK293T cells.

We next investigated evolutionary aspects of the association of ephrin-B1 with HSPGs. HEK293T cells transfected with human, mouse, zebrafish, or *Xenopus* ephrin-B1 cDNA were metabolically labelled with ^35^S-sulfate and lysed. The ephrin-B1 variants were precipitated from the lysates with EphB2-Fc bound to magnetic beads. All the expressed ephrin-B1 variants co-precipitated sulfated macromolecules, although with variable efficiency (supplementary Fig. 1A), corresponding to differences observed for EphB2-Fc binding in flow cytometry analysis of the same cells expressing the different ephrin-B1 variants (supplementary fig. 1B–G). This indicates that the precipitation of ^35^S-labelled HSPGs corresponds to the expression level of each ephrin-B1 variant.

### Deletion of the intracellular last 34 amino acids of ephrin-B1 does not affect association with HSPGs

**Fig. 3:**
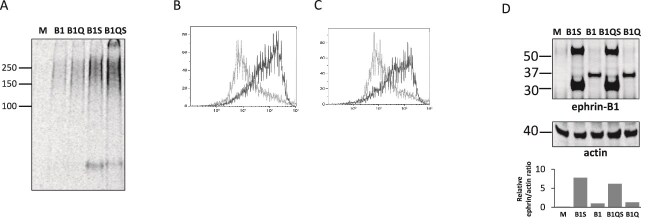
Ephrin-B1 cytoplasmic tail tyrosines are not required for HSPG binding. A) Mock transfected (M) or HEK293T cells expressing ephrin-B1 (B1), ephrin-B1Q (B1Q), ephrin-B1S (B1S), or ephrin-B1QS, using PEI were ^35^S-sulfate labelled, lysed and precipitated with EphB2-fc. B) Flow cytometry of cells expressing ephrin-B1 (grey) or ephrin-B1S (black) incubated with EphB2-fc and PE-antibody. C) Flow cytometry of cells expressing ephrin-B1Q (grey) or ephrin-B1QS (black) after binding of EphB2-fc followed by PE. D) Western blot of lysates from mock (M) transfected and cells expressing human ephrin-B1 (B1), ephrin-B1S (B1S), ephrin-B1Q (B1Q), ephrin-B1QS (B1QS), or mouse ephrin-B1 (B1M). Upper panel detection with anti-ephrin-B1, lower panel with anti-actin for expression level control. Band regions were quantified and relative intensities compared to actin bands and related to lane 3 (insert).

The 34 amino acids at the C-terminal of the intracellular portion of human ephrin-B1 contains six tyrosine residues that may be phosphorylated upon receptor-ligand interaction and in this way involved in intracellular signalling (Kullander and Klein 2002). To investigate whether this region of the cytoplasmic tail is important for the association with HSPGs, we generated an ephrin-B1 construct (ephrin-B1S) lacking these 34 C-terminal (expressing amino acids 1 to 312 of human ephrin-B1). A similar construct was made based on ephrin-B1Q, lacking all SG motifs and the last 34 amino acids, denoted ephrin-B1QS. HEK293T cells were transfected to express these truncated ephrin-B1 variants and subsequently labelled with ^35^S-sulfate overnight, followed by cell lysis. Precipitation with EphB2-Fc from lysates of cells expressing ephrin-B1S and ephrin-B1QS isolated more ^35^S-sulfate-labelled HSPGs that could be isolated from cells expressing ephrin-B1 and ephrin-B1Q (Fig. 3A). EphB2-Fc binding to intact HEK293T cells expressing the same ephrin-B1 variants was studied by flow cytometry analysis. Binding of EphB2-Fc to transfected HEK293T cells displayed similar differences to that observed for precipitation of ^35^S-sulfate-labelled HSPGs from lysates. EphB2-Fc bound much better to cells expressing the truncated ephrin-B1S and ephrin-B1QS variants than to cells expressing the full-length variants ephrin-B1 and ephrin-B1Q (Fig. 3B and C). The cellular levels of these ephrin-B1 variants were also analysed by Western blotting (Fig. 3D) and correlated with the extent of binding of EphB2-Fc to the cell surface of HEK293T cells (Fig. 3B and C). The cells expressing ephrin-B1S or ephrin-B1QS displayed higher levels of these variants than the levels observed for cells expressing wild type ephrin-B1 and ephrin-B1Q. Ephrin-B1S or ephrin-B1QS also showed a tendency to form dimers (Fig. 3D). Thus, deletion of the terminal 34 aa of the cytoplasmic tail of human ephrin-B1 did not abrogate the association with HSPGs (Fig. 3A). The higher cellular levels of the truncated ephrin-B1 variants correspondingly increased the capacity to co-precipitate sulfated HSPGs with EphB2-Fc bound to magnetic beads.

### All ephrin-B members can associate with HSPGs

**Fig. 4:**
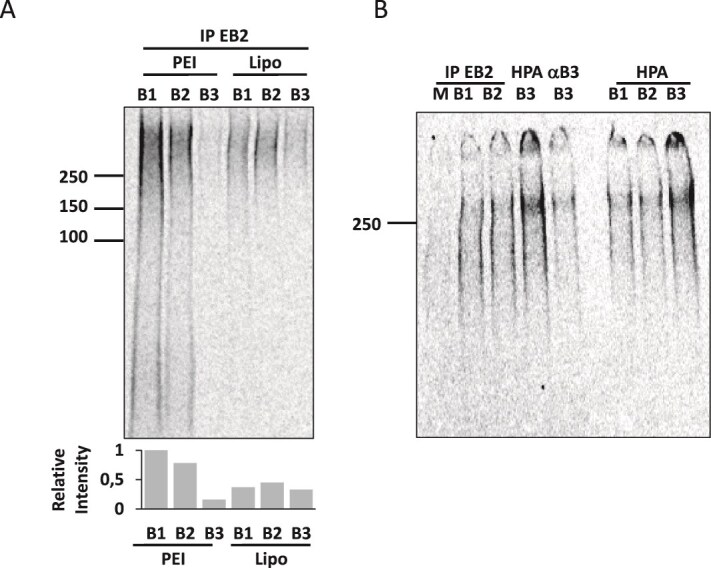
Ephrins-B1, -B2, and -B3 all have the ability to bind HSPGs. A) HEK293T cells transfected to express ephrin-B1 (B1), ephrin-B2 (B2), or ephrin-B3 (B3) using PEI or Lipofectamine (Lipo) were ^35^S-sulfate labelled, lysed, and precipitated with EphB2-fc (EB2). Lanes were quantified by image quant and relative values calculated (insert). B) Cells mock (M) or transfected (Lipo) to express ephrins-B1 (B1), -B2, or -B3, ^35^S-sulfate labelled, lysed, and precipitated with either EB2, HPA (recognizing all ephrin-Bs) or an anti-ephrin-B3 antibody.

HEK293T cells were transfected to express the three different ephrin-B family members (ephrins-B1, -B2, and -B3), using either PEI or Lipofectamine as transfection agent, followed by metabolic labelling with ^35^S-sulfate. EphB2-Fc could precipitate sulfate labelled HSPGs from lysates of both ephrin-B1 and ephrin-B2 expressing cells independently of the transfection agent used, while a weaker signal was observed for cells expressing ephrin-B3 (Fig. 4A). In a recent study we have shown that EphB2-Fc does not bind ephrin-B3 efficiently (Sapkota et al. 2022). We therefore incubated cell lysates with two antibodies that both recognise ephrin-B3. Both antibodies precipitated sulfated macromolecules well from ephrin-B3 transfected cells (Fig. 4B). We have previously shown that the Atlas anti-ephrin-B2 antibody HPA0617188 (Sigma Aldrich) recognises all ephrin-B members in Western blot analysis. Comparable ^35^S signals were observed for all ephrin-B members after precipitating from cell lysates using this antibody (Fig. 4B). This shows that all ephrin-B members, ephrins-B1, -B2, and -B3, can associate with HSPGs at the cell surface.

### Ephrin-B1 interacts with CD44V3–10 and fibroblast growth factor receptors (FGFRs)

**Fig 5:**
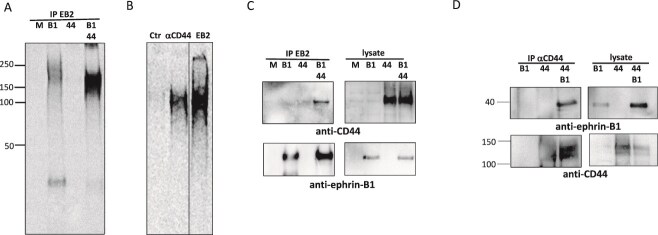
Ephrin-B1 associates with the HSPG CD44V3–10. (A) Mock (M) transfected (Lipo) or HEK293T cells expressing ephrin-B1 (B1), CD44V3–10 (44), or both (B1 44), ^35^S-sulfate labelled, lysed, and precipitated with EphB2-fc. B) Cells co-expressing ephrin-B1 and CD44V3–10 (Lipo) ^35^S-sulfate labelled, lysed, and precipitated with EphB2-fc and protein-G beads and then boiled off the protein-G beads in PBS/0.5% SDS (1/10 of precipitate shown in lane denoted EB2). Remaining sample precipitated with ephrin-B1-fc (Ctr) or α-CD44. C). Lysates from cells transfected as in 5A, but expressing ephrin-B1S were precipitated with EphB2-fc (EB2). Lysates and precipitates were separated by SDS-PAGE and western blotted. Blots were probed with anti-CD44 (upper panel) or anti-ephrin-B1 (lower panel) D) lysates from cells transfected as in 5C precipitated with anti-CD44. Precipitates and lysates separated by SDS-PAGE and western blotted. Blots probed with anti-ephrin-B1 (upper) or anti-CD44 (lower panel).

To identify potential HSPG interaction partners for ephrin-B1, we co-expressed ephrin-B1 and different HSPGs in HEK293T cells, followed by labelling with ^35^S-sulfate and EphB2-Fc mediated precipitation. Co-expressing ephrin-B1 with the HSPG variant of CD44, CD44V3–10, gave an increased signal of ^35^S-sulfated macromolecules when comparing to cells expressing ephrin-B1 alone (Fig. 5A).

To further investigate the nature of the isolated HSPGs, EphB2-Fc bound to magnetic protein G beads was used to precipitate ^35^S-sulfate-labelled HSPGs from lysates co-expressing CD44V3–10 and ephrin-B1, before the precipitated proteins were released from the protein G beads by boiling the samples in lysis buffer with 0.5% SDS. After release, an anti-CD44 antibody was used to precipitate ^35^S-sulfate-labelled CD44V3–10, showing that this HSPG had been co-precipitated with ephrin-B1 and therefore could be an ephrin-B1 interaction partner (Fig. 5B).

We further investigated this potential protein complex by immunoprecipitation and Western blot analysis. In these experiments we expressed ephrin-B1S lacking the C-terminal 34 amino acids of the cytoplasmic tail, since this variant displayed higher cellular levels than the full-length variant (Fig. 3). This could be due to more efficient expression, or reduced endocytosis and degradation of ephrin-B1S. Lysates from transfected cells co-expressing ephrin-B1S and CD44V3–10 were precipitated with EphB2-Fc bound to protein-G beads followed by Western blot analysis. Precipitation from lysates of cells expressing ephrin-B1 alone or ephrin-B1 together with CD44V3–10 showed in both cases reactivity to an anti-ephrin-B1 antibody, while only lysates from cells expressing both ephrin-B1 and CD44V3–10 showed reactivity to an anti-CD44 antibody. This demonstrates that EphB2-Fc can bind to ephrin-B1 also when it is in a complex with CD44V3–10 (Fig. 5C). We also precipitated the same lysates with an anti-CD44 antibody. Also in this case, ephrin-B1 could be co-precipitated with CD44V3–10 (Fig. 5D).

Previous studies have shown that ephrin-B1 can interact with FGFR (Chong et al. 2000; Bong et al. 2004; Moore et al. 2004; Lee et al. 2009). HSPGs are known to present FGFs to FGFRs at the cell surface for productive signalling (Faham et al. 1998; Schlessinger et al. 2000). Other studies have indicated that FGF can bind to CD44 through a HS/heparin-binding domain (Sherman et al. 1998; Jones et al. 2000; Zhen et al. 2018).

**Fig 6:**
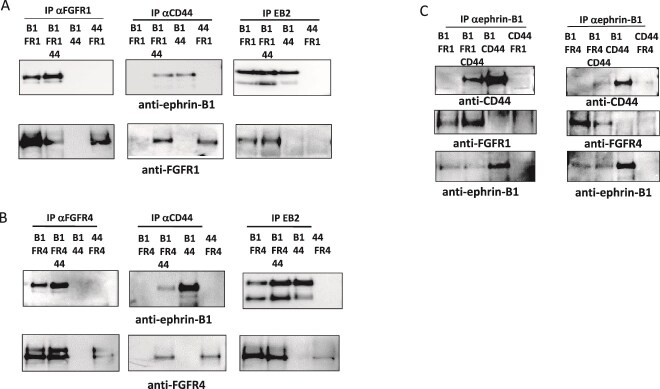
Ephrin-B1 can form a complex with CD44V3–10 and fibroblast growth factor receptors. A) HEK293T cells transfected (Lipo) to express combinations of ephrin-B1S (B1), FGFR1 (FR1), and CD44V3–10 (44). Cell lysates precipitated with anti-FGFR1 (left), anti-CD44 (mid), or EphB2-fc (EB2; right panel), separated by SDS-PAGE and western blotted with anti-ephrin-B1 (upper) or anti-FGFR1 (lower panels). B) Cells were transfected (Lipo) to express combinations of ephrin-B1S (B1), FGFR4 (FR4), and CD44V3–10 (44). Cell lysates precipitated with anti-FGFR4 (left), anti-CD44 (mid) or EphB2-fc (EB2; right panel), separated by SDS-PAGE and western blotted with anti-ephrin-B1 (upper) or anti-FGFR4 (lower panels). C) Cells transfected as in 6A (left) or 6B (right panels) were precipitated with anti-ephrin-B1, separated by SDS-PAGE and western blotted with anti-CD44 (upper), anti-FGFR1, anti-FGFR4 (mid), or anti-ephrin-B1 (lower panels).

We therefore aimed to investigate whether the interaction between ephrin-B1 and CD44V3–10 could also engage FGFRs. By co-transfection of HEK293T cells to express ephrin-B1 and CD44V3–10 together with either FGFR1 or FGFR4, we could show that both FGFR1 and FGFR4 co-precipitated with ephrin-B1 and CD44V3–10 in ternary complexes (Fig. 6A-C). Omitting one of the complex partners from the experiment showed that ephrin-B1 could form binary complexes with CD44V3–10, FGFR1, and FGFR4, and that CD44V3–10 could form binary complexes with FGFR1 and FGFR4.

Precipitation from cell lysates was performed with EphB2-Fc or antibodies against CD44, ephrin-B1, FGFR1 or FGFR4. The precipitates were separated by SDS-PAGE and Western blotted with anti-ephrin-B1, anti-CD44, antiFGFR1, or anti-FGFR4 antibodies. In control experiments, expression of either the protein that was precipitated or the protein that was Western blotted was omitted, to show that the signal was specific. Thus, ephrin-B1 has the ability to form protein complexes with the HSPG CD44V3–10 and FGFRs 1 and 4 (Fig. 6A-C). These complexes can potentially regulate cell surface residency time and turnover of the molecules involved and could in this way also influence signalling properties for these transmembrane proteins.

## Discussion

In the experiments reported here, we show that ephrin-B1 can bind to HSPGs in *cis* in the cell membrane and influence their cellular turnover. The observation was made in HEK293T cells that were transfected to express ephrin-B1, but could also be confirmed in HT-29 cells that express ephrin-B1 endogenously. This finding was consistent across vertebrate species, since expression of ephrin-B1 from zebrafish, xenopus, mice, and humans gave similar results. In fact, all three members of the ephrin-B family, ephrins-B1, -B2, and -B3 can bind HSPGs (in *cis*), as also previously shown for ephrin-B3 (Holen et al. 2011; Prydz et al. 2018), but the mode of binding seems different for ephrin-B1. The difference was observed when transfecting HEK293T cells using PEI as the transfection agent. When comparing to mock transfected cells, and cells expressing ephrin-B2 or ephrin-B3, HEK293T cells expressing ephrin-B1 displayed more HSPGs in the plasma membrane available for binding of soluble ephrin-B3-Fc or anti-HSPG antibodies. HSPGs turnover rapidly by endocytosis (Christianson and Belting 2014). After arriving at the plasma membrane, HSPGs are internalized and their attached GAG chains are cleaved off their protein cores, shortened and eventually degraded in the endocytic pathway (Egeberg et al. 2001; Mani et al. 2007; Adusumalli et al. 2021). PEI is a cationic transfection agent that engages HSPGs in delivery of DNA across the plasma membrane (Mislick and Baldeschwieler 1996) and at the same time induces HSPG endocytosis (Paris et al. 2008; Mozley et al. 2014). The internalization pathway has been described as clathrin- and caveolin-independent, while it depends on dynamin and flotillin-1 (Payne et al. 2007). Endogenous positively charged molecules, like polyamines, are also utilizing HSPGs for internalization (Belting et al. 1999; Cheng et al. 2018), and the influence of ephrin-B1 is therefore of biological interest. The ability of ephrin-B1 to withhold HSPGs at the cell surface does not require the most C-terminal 34 amino acid sequence of the cytoplasmic tail containing tyrosine residues involved in intracellular signalling processes. In fact, the level of cell-associated HSPGs was higher when ephrin-B1 lacking this intracellular portion was expressed in HEK293T cells, in comparison to cells expressing full-length ephrin-B1. Thus, although the presence of ephrin-B1 delays HSPG turnover, there seems to be residual internalization of ephrin-B1 that requires the C-terminal region of ephrin-B1. An alternative possibility could be that removal of the C-terminus of ephrin-B1 reduces the shedding of ephrin-B1 to the extracellular environment (Tomita et al. 2006; Tanaka et al. 2007), but no secretion or shedding of HSPGs to the medium of HEK293T cells could be observed in the experiments reported here (Supplementary fig. 3).

Previous work has shown that both ephrin-A3 (Irie et al. 2008) and ephrin-B3 (Holen et al. 2011) can bind to cell surface HSPGs. Soluble ectodomains of ephrin-A3 and other ephrins were expressed in HEK293T cells. Only ephrin-A3 bound to heparin-Sepharose beads, but ephrin-B3 was not a part of this study (Irie et al. 2008). Ephrin-B3 binding was studied using the extracellular domain of ephrin-B3 fused to the Fc portion of mouse IgG2b (Holen et al. 2011; Prydz et al. 2018). This fusion protein could bind to both soluble and cell surface HSPGs, and this binding was dependent on the basic amino acids arg178 and lys179 in the juxtamembrane region of ephrin-B3. Similar fusion proteins of the other ephrin-B family members showed some (ephrin-B2) or little (ephrin-B1) binding to immobilized heparin in this study, while ephrin-A1 and ephrin-A3 also showed some heparin binding in the same study (Holen et al. 2011). While ephrin-B2 has a positively charged amino acid (lys177) that could contribute to heparin binding, the soluble ephrin-B1 ectodomain could better bind heparin after transfer by site-directed mutagenesis of two basic amino acids corresponding to those required for heparin binding in ephrin-B3 (Holen et al. 2011). In a microarray based study, both human ephrin-A1 and mouse ephrin-A5 were shown to bind to heparin and heparan sulfate (Shipp and Hsieh-Wilson 2007). In an unpublished study, we metabolically labelled cultured mouse brain slices with ^35^S-sulfate lysed and incubated lysates with FC-fusion proteins of ephrin-A1, ephrin-A3, and ephrin-B1. Only ephrin-B1 of the three tested ephrins could bind HSPGs in these lysates (Prydz et al. unpublished). Thus, there are discrepancies among studies of binding of ephrins to HSPG. Most, if not all, studies have been conducted with commercially available soluble ephrin-Fc fusion proteins. All together, the differences observed indicate that the HS sulfation pattern plays a role in ephrin binding, but some discrepancies can only be resolved by further experiments.

Previously obtained data from our own experiments suggested that such soluble Fc-fusion proteins of ephrin-B2 and -B3, but not ephrin-B1 could bind to heparin or HSPGs on human cell lines, representing binding in *trans*, while membrane associated ephrin-B1 (and also ephrin-B2 and ephrin-B3) in the present study is shown to bind HSPGs in *cis* laterally in the cell membrane. These ephrins have a high degree of sequence similarity, but the sequence from amino acid 168 to amino acid 236 is unique to ephrin-B1 and could be important in this respect (Supplementary Fig. 2).

Soluble ephrin-B3-Fc was previously shown to be able to bind HS chains attached to a number of PG protein cores, including CD44V3–10, an HSPG splice variant of CD44. When co-expressing full-length ephrin-B1 and CD44V3–10 in HEK293T cells, these were shown to bind one another by co-precipitation in combination with Western blotting. The complex could be isolated using antibodies to CD44 or ephrin-B1, and in addition with the ephrin receptor EphB2. The interaction between CD44V3–10 and ephrin-B1 is to our knowledge the first report of an interaction between any variant of CD44 and ephrin-B1. Both ephrin-B1 (Chong et al. 2000; Moore et al. 2004; Poliakov et al. 2008; Lee et al. 2009) and CD44 (Nedvetzki et al. 2003; Zhen et al. 2018) have previously been reported to interact with FGF and/or FGFRs. We therefore addressed whether the ephrin-B1/CD44V3–10 complex could take on an additional binding partner. FGFR1 and FGFR4 could both be co-isolated with ephrin-B1 and CD44V3–10 separately and also together in a ternary complex, using anti-FGFR antibodies, antibodies directed to CD44 or ephrin-B1, or by using the EphB2 ephrin-B receptor. The interaction of ephrin-B1 with FGFRs 1 and 4 can occur in the absence and presence of CD44V3–10 expression and is therefore not dependent on HS chains attached to this protein core but could still employ endogenously expressed HSPGs. The ephrin-B1 variant expressed was ephrin-B1S, lacking the 34 most C-terminal amino acids that contain tyrosines important for intracellular signalling and recruitment of cytoplasmic proteins. Interestingly, mutations in the genes encoding FGFR2, FGFR3, and ephrin-B1 can all lead to craniosystinosis that is defined as premature fusion of the cranial sutures (Johnson and Wilkie 2011). The tyrosine kinase ErbB2 can also associate with ephrin-B1 and Src in a signalling complex that interacts with EphB2 (Vermeer et al. 2012). While the HSPG Syndecan-1 has been implicated as a co-receptor for ErbB2, the role of HS was not addressed for this complex.

HSPGs have been shown to be required for proper FGF signalling via FGFRs (Chen et al. 2023). HSPGs both at the cell surface and in the extracellular matrix can function as reservoirs of bound FGFs. An increase in the cell surface amount of HSPGs induced by ephrin-B1 could indirectly influence the signalling strength by providing more ligand for the receptors, but possibly also by prolonging the duration of the signalling.

Cell surface HSPGs are either transmembrane proteins, as CD44V3–10 and members of the Syndecan-family of HSPGs (Hassan et al. 2021), or attached to the plasma membrane via a GPI lipid anchor, like the members of the Glypican family of HSPGs (Fransson et al. 2004). A large fraction of cell surface HSPGs is internalized via a flotillin-dependent pathway (Payne et al. 2007). Flotillins localize to cholesterol-rich lipid rafts in the plasma membrane which are enriched in both flotillins and caveolins—proteins that promote clathrin-independent endocytic mechanisms. Syndecans have been reported to interact with flotillin-1 (Chen et al. 2018), while glypicans localize to lipid rafts via their GPI-anchor (Taylor et al. 2009). Also CD44 has been shown to localize to lipid rafts in a cholesterol-dependent manner (Murai et al. 2011), while ephrin-B1 can bind to caveolin-1 (Tiruppathi et al. 2020). Thus, ephrin-B1 may colocalize with CD44V3–10 and other HSPGs in cholesterol-rich plasma membrane domains. To what extent these molecules form higher order complexes and how they reciprocally influence each other’s biology is a topic of further studies. Syndecan-2 has, however, been suggested to play a role in the clustering of ephrin-Bs and their EphB receptors (Irie and Yamaguchi 2004).

To conclude, we have shown that all ephrin-Bs can interact in *cis* with HSPGs in the cell membrane when expressed in HEK293T cells, while only ephrin-B1 delays the PEI-induced internalization of HSPGs. When co-expressed with CD44V3–10 and FGFRs 1 or 4, ephrin-B1 forms complexes with these transmembrane proteins individually or jointly with both CD44V3–10 and FGFRs.

## Materials and methods

### Fusion proteins and antibodies

The fusion proteins ephrin-B3-Fc and EphB2-Fc were purchased from R&D Technology. The following antibodies were used for immunoprecipitation and Western blot analysis: anti-CD44V3-V10 (R&D Technology), anti-FGFR1 and anti-FGFR4 (Cell signalling), anti-ephrin-B1 (A20, Santa Cruz Biotechnology), anti-ephrin-B1/B2/B3 (C18, Santa Cruz Biotechnology), anti-ephrin-B2 (Santa Cruz Biotechnology), anti-ephrin-B3 (Santa Cruz Biotechnology), anti-heparan sulfate (10E4; Amsbio), anti-ephrin-B1 (HPA0617188, Sigma Aldrich). Anti-mouse-HRP and anti-rabbit-HRP antibodies (Jackson ImmunoResearch) were used for Western blot detection.

### Cells and constructs

HEK293T and HT-29 cells were incubated in high-glucose DMEM, with L-glutamine, 10% (v/v) heat-inactivated FBS (PromoCell), and 1% penicillin/streptomycin (PAA Laboratories) at 37 °C in humidified atmosphere with 5% CO_2_. Expression vectors with cDNAs encoding PGs tested; Agrin (membrane bound, accession no. NM_198576), Perlecan, and CD44V3–10 were purchased from Origene (US), while Collagen XVIII cDNA was from Source BioScience (UK). The expression clones of Syndecans, human ephrin-B1, ephrin-B2, and ephrin-B3 have been described previously (Prydz et al. 2018). Ephrin-B1 cDNA from *Xenopus* was a kind gift from Professor Ira Daar, Cancer & Developmental Biology Laboratory, National Cancer Institute, MD, USA. Ephrin-B1 mouse expression vector was from SINO Biological. FGFR1 and FGRF4 in pcDNA3 was a kind gift from Dr J. Wesche, The Norwegian Radium Hospital, Oslo, Norway. We made several constructs and all primers used in this study are described in Table 1.

**Table 1:** Primers used in this study.

**Primer**	**Sequence (5′–3′)**
**Cloning of *efnb1***	
Ephrin-B1_Zebra_For	GATCGGTACCCCGCTCTGTTGCATGTGG
Ephrin-B1_Zebra_Rev	GATCTCTAGATTATTGCGCTTGCTTGGCTC
**Site directed mutagenesis of EFNB1**	
S46A-For	CCTGGCTGGGAAGGGCTTGGTG
S46A-Rev	CCCAGCCAGGAACTTGGGGTTG
S226/227-For2	GGGGCGCTGCTGGGGACCCTGATGGC
S226/227-Rev2	TCCCCAGCAGCGCCCCCACTTGCACCTG
S218/223A-For	GCAGGCCCAGGTGCAGCAGGGGGCGCTGCTGGG
S218/223A-Rev	TGCTGCACCTGGGCCTGCCTTCTCTTCCTGGTTCACA
**3′-truncation of EFNB1**	
EB1_trunc_For_N	CAGAGAACAACTGAGGATCCACTAGTAACG
EB1_trunc_Rev_N	GATCCTCAGTTGTTCTCTGTAGTCCGT
**Control primers for verification of mutations**	
EphrinB1-For	ATGGCTCGGCCTGGGC
S226/227-C-Rev	CAGGGTCCCCAGCAGC
S218/223-C-Rev	GCTGCACCTGGGCCTG
EB1_trunc_C_Rev	ACTAGTGGATCCTCAGTTG
S46A-C-For	CAACCCCAAGTTCCTGGC
EphrinB1Var-Rev	CTTGGAGTTGAAGAAGCCAT
**Sequencing primers**	
T7	TAATACGACTCACTATAGGG
Sp6	CATTTAGGTGACACTATAG

### Cloning of efnb1 from zebrafish

The primers Ephrin-B1_Zebra_For and Ephrin-B1_Zebra_Rev were used to amplify *efnb1* from cDNA generated from mRNA isolated from zebrafish (a kind gift from Professor Anne Simonsen, Department of Medicine, University of Oslo, Norway). The *efnb1* amplicon was cloned into pcDNA3 between the KpnI and XbaI restriction sites using high fidelity versions of the restriction enzymes and the rapid DNA ligation kit (Thermo Fisher Scientific, Waltham, MA, USA) according to the manufacturer’s instructions. DNA was introduced into chemo competent *Escherichia coli* MC1061. Bacteria were plated on LB-plates supplemented with ampicillin (25 μg/mL) and tetracycline (10 μg/mL). Colonies were screened for constructs by colony PCR using the primers used for cloning. DNA was extracted from colonies giving rise to amplicons of correct size, and sequenced at Eurofins Genomics Germany GmbH (Ebersberg, Germany) using primers T7 and SP6.

### Mutagenesis of ephrin-B1

The Ephrin-B1 construct carrying the human ephrin B1 gene under control of the CMV promoter was used as template for overlap extension site directed mutagenesis of ephrin-B1. Five mutations were introduced into ephrin-B1, which altered the serines in positions 46, 218, 223, 226, and 227 to alanine. This was done in three steps: First, the mutations introducing S226A and S227A were produced using primers S226/227-For2 and S226/227-Rev2. This construct was used as template to introduce the mutations giving S218A and S223A, using primers S218/223-For and S218/223-Rev. Finally, the construct with S218A, S223A, S226A, and S227A was used as template to make the mutation introducing the S46A alteration. The resulting construct was referred to as Ephrin-B1Q. Following each mutagenesis step the amplicons were treated with DpnI-HF (Thermo Fisher Scientific) according to the manufacturer’s instructions, and DNA was introduced into chemo competent *E. coli* MC1061. Bacteria were plated on LB-plates supplemented with ampicillin (25 μg/mL) and tetracycline (10 μg/mL). Colonies were screened for mutated constructs by colony PCR using primers: S46A-C-For and EphrinB1Var-Rev (mutation introducing S46A), EphrinB1-For and S218/223-C-Rev (S218A, and S223A) and EphrinB1-For and S226/227-Rev (S226A, S227A). DNA was extracted from colonies giving rise to amplicons of correct size, and sequenced at Eurofins Genomics Germany GmbH (Ebersberg, Germany) using primers T7 and SP6.

### Truncation of ephrin-B1 and ephrin-B1Q

Overlap extension PCR was carried out to produce C-terminally truncated ephrin-B1 and ephrin-B1Q, using primers EB1_trunc_For_N and EB1_trunc_Rev_N together with the templates pEphrin-B1 or Ephrin-B1Q. Amplicons were treated with DpnI and the DNA was introduced into chemo-competent *E. coli* MC1061. Bacteria were plated on LB plates supplemented with ampicillin (25 μg/mL) and tetracycline (10 μg/mL). Colonies were screened for truncations by colony PCR using primers EphrinB1-For and EB1_trunc_C_Rev. DNA was extracted from colonies giving rise to amplicons of correct size, and sequenced at Eurofins Genomics Germany GmbH (Ebersberg, Germany) using primers T7 and SP6. The truncated constructs were named ephrin-B1S and ephrin-B1QS.

### Flow cytometry analysis

Cells were transfected with PEI (Huh et al. 2007) or Lipofectamine to express variants of ephrin-B family members. The cells were then suspended in PBS, 1% BSA and incubated with ephrin–B3-Fc (30 μg/mL), EphB2-Fc fusion proteins (30 μg/mL), or the 10E4 anti-heparan sulfate antibody (10 μg/mL) for 30 min at 4 °C before staining with phycoerythrin (PE) conjugated anti-human IgG (Jackson ImmunoResearch) or with FITC conjugated anti-mouse IgM (Thermo Fisher Scientific). Cells were then analysed with a FACSort flow cytometer (BD Biosciences), flow data were collected with CellQuest 3.3 (BD Biosciences) and analysed using the Kaluzo program (BD Biosciences). At least 20,000 cells gated as viable were analysed per sample.

### ^35^S-sulfate metabolic labelling of proteoglycans

HT-29 cells or transfected HEK293T cells were incubated overnight two days after transfection in sulfate-free RPMI 1640 medium with 2% FBS and 0.2 mCi/ml of ^35^S-sulfate (Hartmann Analytic). Media were removed for analysis of secreted macromolecules, and the cells were washed two times 15 min in cold PBS, then lysed in 0.5% Nonidet P40 (NP40) in PBS with protease inhibitors (Sigma–Aldrich)] for 30 min before clearing by centrifugation (18,000 g for 5 min at 4 °C). EphB2-Fc (1 μg), anti-ephrin-B1, or anti-CD44 was added to cell lysates, before incubation with agitation for 3 h at 4 °C. Protein G beads (10 μL; Thermo Fisher Scientific) were added before incubation for another 1 h. The precipitates were washed 3 times with PBS and then loaded onto SDS-PAGE gels. The gels were fixed (30 min), treated with Amplify (GE Healthcare Biosciences), and dried, followed by analysis in a Typhoon 9400 or STORM 860 phosphorimager, using ImageQuant (GE Healthcare).

### Analysis of the effect of ephrin-B1 expression on HSPG shedding from HEK293T cells

Untransfected and HEK293T cells transfected to express either CD19 (transfection control) or ephrin-B1 were metabolically labelled with ^35^S-sulfate overnight and the media were collected and centrifuged to remove unattached cells. Labelled macromolecules in the medium supernatants were separated from free ^35^S-sulfate on Sephadex G50 fine gel filtration columns. Each sample was divided in two equal portions and one of these was treated with chondroitinase ABC to degrade secreted chondroitin sulfate PGs (Vuong et al. 2006; Dick et al. 2008) while the other half was left untreated. The samples were then analysed by SDS-PAGE and prosessed and analysed as described above.

### Protein precipitation and western blot analysis

Transfected HEK293T or HT-29 cells were lysed in 0.5% NP40 in PBS, supplemented with protease inhibitors. Ephrin-B1, CD44V3-V10, and FGFR-1 and -4 were precipitated from cell lysates (diluted once in PBS) with specific antibodies (0.5–1 μg) and protein-G beads for 2 h before 3 washes in cold PBS. EphB2 binding proteins were precipitated with EphB2-Fc. Precipitates and cell lysates were separated in ready-cast SDS-polyacrylamide 4–12% gradient gels (NuPAGE or Bio-Rad Laboratories) and transferred onto nitrocellulose membranes (Whatman). The membranes were incubated for 1 h with primary antibody at room temperature or overnight at 4 °C. Membranes incubated with unconjugated primary antibodies were washed and incubated with the appropriate HRP-linked secondary antibody for 1 h at room temperature. Antibody binding was detected using the ECL (enhanced chemiluminescence) Plus Western blotting detection system (GE Healthcare). Membranes were stripped in 2% (w/v) SDS [62.5 mM Tris/HCl (pH 6.8) and 0.1 mM 2-mercaptoethanol] at 55 °C for 30 min when incubated sequentially with more than one antibody.

## Supplementary Material

Figure_legends_for_supplementary_figures_GLYCO-2024-00018_R2_cwaf020

Supplementary_figure_1_GLYCO-2024-00018_R1_cwaf020

Supplementary_figure_2_GLYCO-2024-00018_R1_cwaf020

Supplementary_figure_3_GLYCO-2024-00018_R1_cwaf020

## Data Availability

The data are available on request from corresponding authors.
