# Correction to: Ephrin-B1 regulates cell surface residency of heparan sulfate proteoglycans (HSPGs) and complexes with the HSPG CD44V3–10 and fibroblast growth factor receptors

**DOI:** 10.1093/glycob/cwaf040

**Published:** 2025-07-09

This is a correction to: Kristian Prydz, Roger Simm, Erna Davydova, Hans-Christian Aasheim, Ephrin-B1 regulates cell surface residency of heparan sulfate proteoglycans (HSPGs) and complexes with the HSPG CD44V3–10 and fibroblast growth factor receptors, *Glycobiology*, Volume 35, Issue 6, June 2025, cwaf020, https://doi.org/10.1093/glycob/cwaf020

The originally published version of this manuscript contained the following errors:

1. The supplementary figures were erroneously omitted

2. Author Kristian Prydz was not designated as a co-corresponding author

3. The affiliation of author Hans-Christian Aasheim was incorrect

The publisher apologizes for these errors, which have been corrected.

## Supplementary Material

Figure_legends_for_supplementary_figures_GLYCO-2024-00018_R2_cwaf020

Supplementary_figure_1_GLYCO-2024-00018_R1_cwaf020

Supplementary_figure_2_GLYCO-2024-00018_R1_cwaf020

Supplementary_figure_3_GLYCO-2024-00018_R1_cwaf020

