# Supplementary material for: Ephrin-B1 regulates cell surface residency of heparan sulfate proteoglycans (HSPGs) and complexes with the HSPG CD44V3–10 and fibroblast growth factor receptors
Source: Glycobiology. 2025 Apr 28;35(6):cwaf020. doi: 10.1093/glycob/cwaf020 (PMC12036661; doi:10.1093/glycob/cwaf020)
Supplement: Figure_legends_for_supplementary_figures_GLYCO-2024-00018_R2_cwaf020 [file figure_legends_for_supplementary_figures_glyco-2024-00018_r2_cwaf020.docx]

**Figure legends for supplementary figures GLYCO-2024-00018.R2**

**Supplementary figure 1. Ephrin-B1 from vertebrates can bind HS proteoglycans.**

(A) Mock (M), human ephrin-B1 (B1), mouse ephrin-B1 (B1M), zebrafish ephrin-B1 (B1Z), Xenopus ephrin-B1 (B1X), and human ephrin-B1S (lacking the C-terminal part of the cytoplasmic tail) were transiently expressed in HEK293T cells. Cells were then metabolically labelled overnight with ^35^S-sulfate and lysed. Comparable amounts of each cell lysate were precipitated with EphB2-Fc bound to magnetic beads. (B) Flow cytometry analysis of mock transfected cells after incubation with EphB2-Fc (grey) and PE-antibody second layer. Black histogram is irrelevant control (PE-second layer only). (C) Flow cytometry analysis of mock (grey) and human ephrin-B1 (black) transfected cells after incubation with EphB2-Fc (black) and PE-second layer. (D) Flow cytometry analysis of mock (grey) and mouse ephrin-B1 (black) transfected after incubation with EphB2-Fc (black) and PE-second layer. (E) Flow cytometry analysis of mock (grey) and zebrafish ephrin-B1 (black) transfected cells after incubation with EphB2-Fc (black) and PE-second layer. (F) Flow cytometry analysis of mock (grey) and *Xenopus* ephrin-B1 black) transfected cells incubation with EphB2-Fc (black) and PE-second layer. (G) Flow cytometry analysis of mock (grey) and human ephrin-B1S (black) transfected cells after incubation with EphB2-Fc (black) and PE-second layer.

**Supplementary figure 2. Sequence comparison of ephrin-Bs.**

Alignment of the amino acid sequences of human ephrin-B1, -B2, and -B3.

**Supplementary figure 3. Analysis of shedding of HSPGs from HEK293T cells.**

Untransfected (lanes 5-8) or HEK293T cells transfected to express CD19 (lanes 1 and 2) transfection control) or ephrin-B1 (lanes 3 and 4) were metabolically labelled with ^35^S-sulfate overnight. Medium samples were treated (lanes 2,4,6,8) or not (lanes (1,3,5,7) with chondroitinase ABC to degrade chondroitin sulfate PGs and analyzed by SDS-PAGE. No HSPGs have been shed or secreted from the HEK293T cells in the absence or presence of ephrin-B1 expression. The gel shows one representative of three experiments.
