# Supplementary figures and images for: Ephrin-B1 regulates cell surface residency of heparan sulfate proteoglycans (HSPGs) and complexes with the HSPG CD44V3–10 and fibroblast growth factor receptors
Source: Glycobiology. 2025 Apr 28;35(6):cwaf020. doi: 10.1093/glycob/cwaf020 (PMC12036661; doi:10.1093/glycob/cwaf020)

Supplementary Fig 1

A

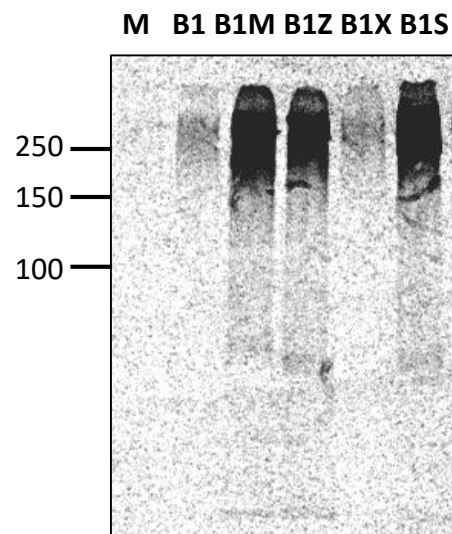

B

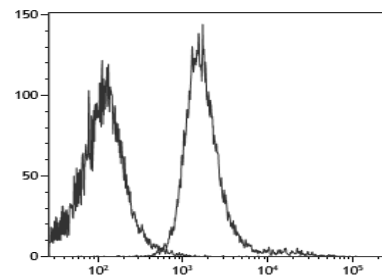

C

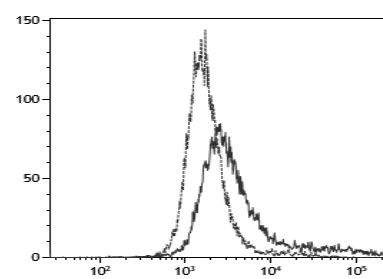

D

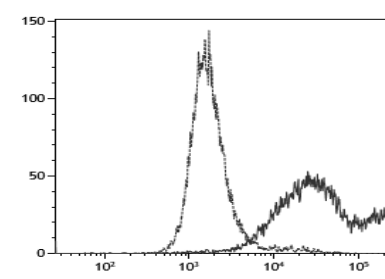

E

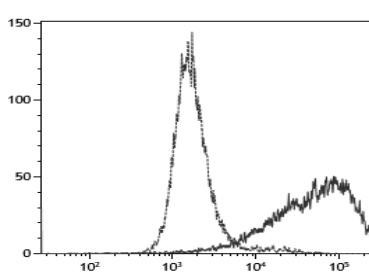

F

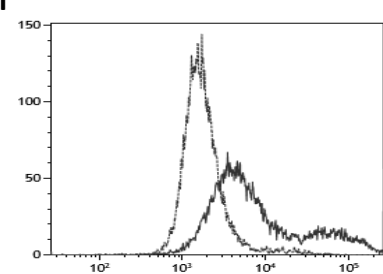

G

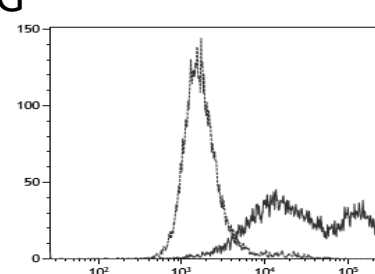

Supplement: Supplementary_figure_1_GLYCO-2024-00018_R1_cwaf020 [file supplementary_figure_1_glyco-2024-00018_r1_cwaf020.pdf]

Supplementary Fig 3

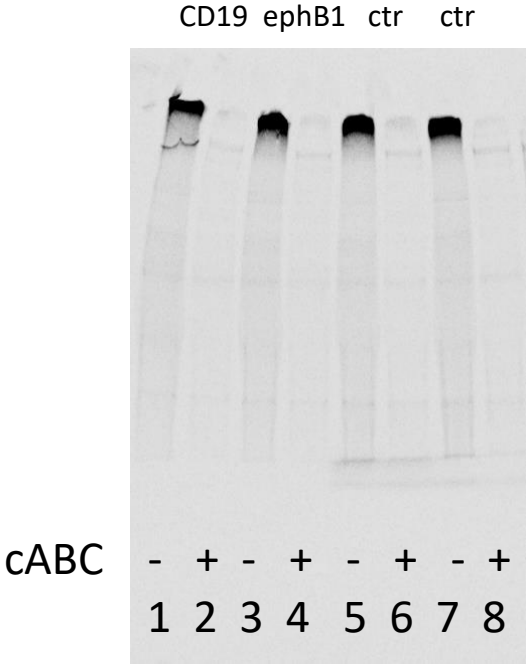

Supplement: Supplementary_figure_3_GLYCO-2024-00018_R1_cwaf020 [file supplementary_figure_3_glyco-2024-00018_r1_cwaf020.pdf]
