# Supplementary material for: Ephrin-B1 regulates cell surface residency of heparan sulfate proteoglycans (HSPGs) and complexes with the HSPG CD44V3–10 and fibroblast growth factor receptors
Source: Glycobiology. 2025 Apr 28;35(6):cwaf020. doi: 10.1093/glycob/cwaf020 (PMC12036661; doi:10.1093/glycob/cwaf020)

Supplementary Fig 2

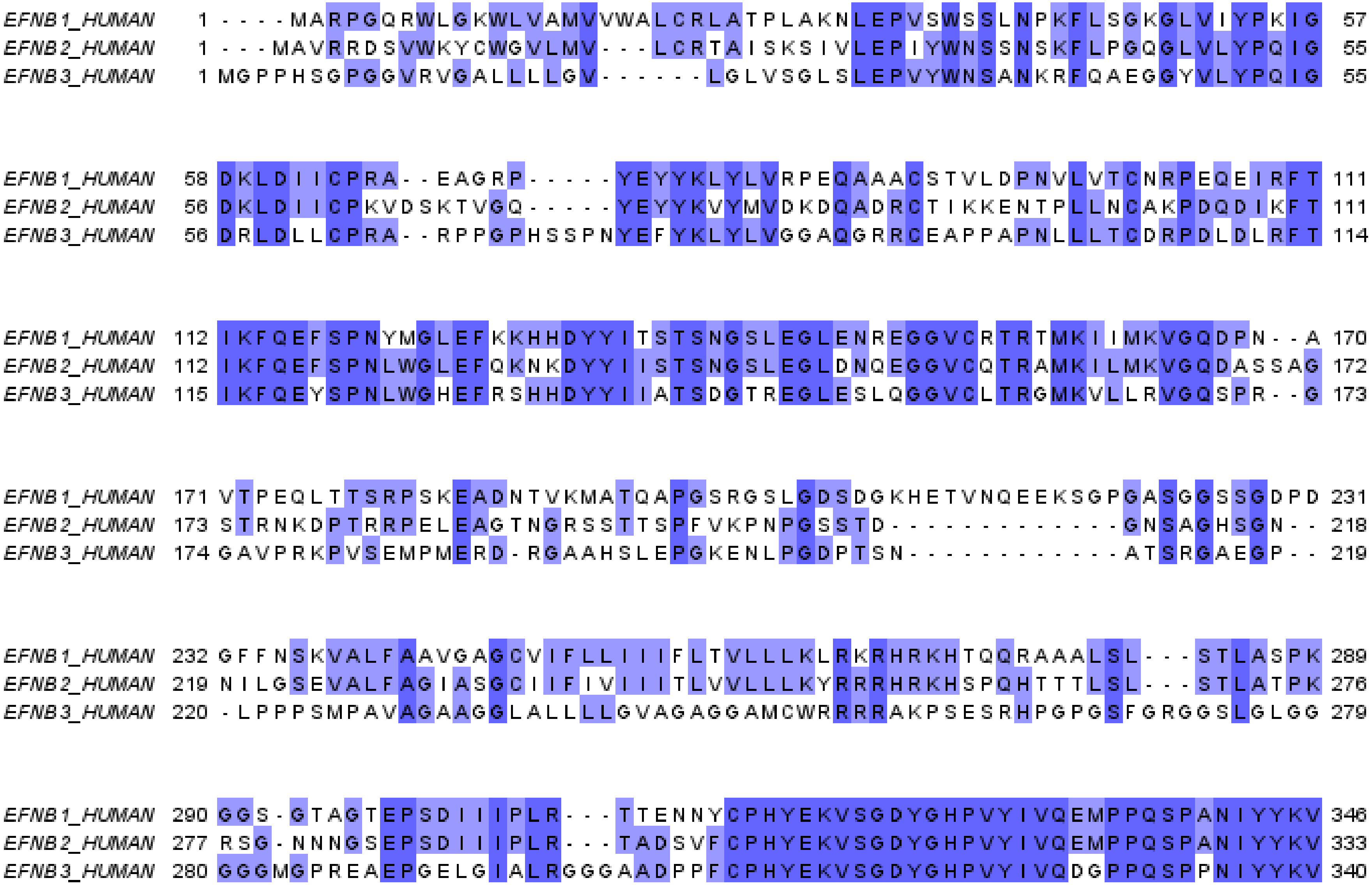

Supplement: Supplementary_figure_2_GLYCO-2024-00018_R1_cwaf020 [file supplementary_figure_2_glyco-2024-00018_r1_cwaf020.pdf]
